# Death in Community Australian Football: A Ten Year National Insurance Claims Report

**DOI:** 10.1371/journal.pone.0159008

**Published:** 2016-07-28

**Authors:** Lauren V. Fortington, Caroline F. Finch

**Affiliations:** Australian Collaboration for Research into Injury in Sport and its Prevention (ACRISP), Federation University Australia, PO Box 668, Ballarat, 3353, Victoria, Australia; University of Sydney, AUSTRALIA

## Abstract

While deaths are thought to be rare in community Australian sport, there is no systematic reporting so the frequency and leading causes of death is unknown. The aim of this study was to describe the frequency and cause of deaths associated with community-level Australian Football (AF), based on insurance-claims records. Retrospective review of prospectively collected insurance-claims for death in relation to community-level AF activities Australia-wide from 2004 to 2013. Eligible participants were aged 15+ years, involved in an Australian football club as players, coaches, umpires or supporting roles. Details were extracted for: year of death, level of play, age, sex, anatomical location of injury, and a descriptive narrative of the event. Descriptive data are presented for frequency of cases by subgroups. From 26,749 insurance-claims relating to AF, 31 cases were in relation to a death. All fatalities were in males. The initial event occurred during on-field activities of players (football matches or training) in 16 cases. The remainder occurred to people outside of on-field football activity (n = 8), or non-players (n = 7). Road trauma (n = 8) and cardiac conditions (n = 7) were the leading identifiable causes, with unconfirmed and other causes (including collapsed or not yet determined) comprising 16 cases. Although rare, fatalities do occur in community AF to both players and people in supporting roles, averaging 3 per year in this setting alone. A systematic, comprehensive approach to data collection is urgently required to better understand the risk and causes of death in participants of AF and other sports.

## Background

A physically active lifestyle associated with sport and recreation activities is strongly encouraged for its positive health benefits. Despite the efforts of governments, health professionals and sports bodies toward risk management and creating safe sporting environments, injury and illness do occur, including those that result in death. In a sports setting, death can be classified as being directly or indirectly related to sports activity. Fatalities that are directly related to sport result during actual sport participation or execution of the fundamental skills of the sport. Indirect fatalities comprise underlying systemic failures or existing disease that occur during participation or are in some way exacerbated through the player’s participation, usually being cardiac or respiratory in nature.[1] Sporting teams and clubs can also be impacted when a death is unrelated to direct participation in the sport but is instead linked to a club supporting role (coach, trainer), social event or during travel to and from club activities.[2] Understanding the leading causes of deaths in different sporting environments is an important step towards providing appropriate emergency management plans and the possible prevention of fatalities.[3]

Australian football (AF) is played nationwide in Australia by over 1.2 million male and female participants of all ages, and many more people taking part through support roles including umpiring, coaching and management.[4] The benefits of being an active member of an AF club, whether as a player, coach, official or volunteer, are associated with positive health outcomes, as well as strong interpersonal relationships between members and economic/social advantages brought to local communities.[5] While deaths are generally thought to be rare in the setting of community AF, and indeed most other sports, there is no currently no systematic reporting of deaths in any Australian sport so the frequency and leading causes are unknown.

Insurance claims have been proposed as a valuable data source for injury surveillance in sport owing to the ongoing and systematic approach to data collection by the companies who provide insurance coverage.[6, 7] By their nature, insurance data tends to capture injuries at the most severe end of the injury spectrum, particularly those requiring medical treatment or impacting an individual financially. This coverage makes insurance data a potentially good source for claims relating to deaths as participants’ next of kin may be likely to seek compensation to help manage any medical/support costs.

The aim of this paper is to report the number and cause of deaths relating to community-level AF in Australia that were reported to a nationwide AF insurance agency, over a ten year period from 2004–2013. This is important because, with an understanding of the number of fatality events that occur and the leading causes of these events, key areas of interest can be identified and measures for prevention developed and monitored.

## Methods

The Federation University Human Research Ethics Committee provided approval for investigation of the de-identified data extracted for this study (E13-016).

This study is a review of prospectively collected data from the Australian Football National Risk Protection Program (AFNRPP). The AFNRPP provides personal accident cover (including injury and death) through the insurance agency JLT (Jardine Lloyd Thompson) Sport to all registered community AF club members.[8] The ‘insured’ is represented by the association/leagues in which clubs participate, with over 260 leagues consistently since 2004. The ‘insured persons’ includes all players, officials, volunteers, trainers, runners, club and league appointed umpires, coaches, directors, officers, committees, sub-committees, regional boards and work experience students. The number of insured persons would expect to vary substantially at each club, with no specific numbers available.

The policy reimburses a portion of non-Medicare (standard national government coverage) medical costs and in some cases (those with an extended policy), loss of income for up to 12 months. All football-related events and activities are covered; this means claimants do not necessarily have to be playing football (games/training) at the time of the injury/death and the injury/death data is not only reported for players. Claims are submitted up to 180 days from the date of a claimable event by the player, club or next of kin.

All claims for injury (including death) are reported via a standardised insurance claim form.[8] The form is completed by the claimant, the football club president and a medical practitioner. Data from this form is subsequently entered into the insurer’s database by an administrator.

For this study, insurance claims with the nature of injury reported as ‘death’ were extracted. Details extracted for all cases included: injury year, level of play (i.e. senior or junior league), activity at time of event (playing, training, travelling, coaching, etc), age, sex, and a text narrative description of the event. Using the text narrative description, a cause was identified where available (as cardiac-related, road trauma, unconfirmed or other). A conservative approach was taken so all cases that did not have a clearly reported diagnosis were labelled as unconfirmed. Data were included from 1 January 2004 to 31 December 2013, inclusive.

A descriptive analysis is presented with the frequency of cases reported in different subgroups. To ensure the privacy of individual player data reported, details are not reported for categories with fewer than five cases. For this reason, all data are reported across the entire period and analysis of time trends was not possible. Incidence rates of death by club-participation estimates were calculated for players (number of player-related deaths divided by all participants over the ten year period,) with a 95% confidence interval (95% CI). Participation data were extracted for club participation from the AFL Annual Reports published online for 2006–2013.[9] As no equivalent data were identified for 2004 or 2005, participation numbers from 2006 were substituted. This may have led to a slight overestimate of club participation numbers in these years as there was annual growth in overall participation (inclusive of total numbers, schools, other competitions) for each successive year. Club participation numbers include youth and adult ages. While the majority are male club participants, there is a small, but growing, proportion of females which will have impacted on increased numbers in later years (i.e. particularly since 2010). As only players were included in the denominator (not people in other roles), we correspondingly limited the numerator (number of deaths) to players only. Analyses were performed using Microsoft Excel and OpenEpi.com.

## Results

From a total of 26,749 AF-related injury claims to the AFNRPP, 31 cases were for death. This represents an average of 3 deaths per year over the ten year period.

All cases were males, with the majority occurring on-field in football matches/training (n = 18). Most claims were for players (n = 24), mainly during football matches/training (n = 16). Five cases involved players aged 15–17 years, the remainder of cases were aged 18+ years (n = 26). Seven claims were for people in other football roles (non-player roles).

Cause was identified as road trauma (n = 8), cardiac (n = 7) and unconfirmed/other (n = 16) (Table 1). Of the other/unconfirmed cases, n = 8 had a descriptive narrative which included ‘collapsed,’ n = 5 had no indication of what occurred (i.e. narrative essentially stated simply ‘death’) and n = 3 had a cause that could be confirmed from the narrative as not being linked to cardiac or road trauma (but reason not disclosed due to privacy.) Of the road trauma, no cases involved multiple fatalities (i.e. one player died in each event.)

**Table 1:** Details of 31 cases of death relating to community level Australian Football club players, support staff and volunteers from 2004–2013 reported to a nationwide insurance agent.

	n	%
**Who/activity**		
Player participating in football match or training	16	52
Player involved in other/unknown activity (travel, social activity)	8	26
People in non-player roles	7	23
**Cause**		
Road trauma	8	26
Cardiac	7	23
Unknown/other^a^	16	52
**Location**^b^		
Initial event occurred on-field	18	58
Initial event occurred off-field	10	32
Unknown	3	10
**Total**	**31**	

^a^a number of cases listed as ‘collapsed’ or similar in the descriptive narrative and were referred to the coroner

^b^ location of the initial event that led to death, person may have subsequently died in hospital

The incidence rate of death was estimated for the 24 players, over a total participation number of 2,965,122 players from 2004–2013. The incidence rate for player-deaths was 0.8/100,000 club-participants (95%CI: 0.5; 1.2)

## Discussion

Reporting deaths in sport is an important starting point toward improved emergency management and safety for players. In the United States, a long history of catastrophic event data collection has demonstrated positive impacts for participants through rule changes, equipment modifications and emergency service awareness/preparedness.[1, 10] This is the first study to report all-cause deaths from Australia-wide insurance claims in any sport in Australia. The data confirms that while deaths in community AF settings are rare, they do occur, with the average number of deaths occurring at 3 per year nationally. Half of the fatalities were in players from initial incidents occurring on the football field. The limitations in the data support the need for a systematic approach to data collection, following a best practice process [11], to better understand why these events occur.

Key issues relating to both on-field (cardiac and unknown/other causes) and off-field deaths are discussed below in relation to previous studies in AF and other sports.

## On-field deaths

The only category for on-field incidents leading to death that could be identified from the data was ‘cardiac-related’. The large number of cases that could only be assigned to ‘unconfirmed/other’ as cause, but with descriptions of ‘collapsed’ in the text narratives, suggest the data are likely to underestimate the number of cardiac-related deaths. Sudden cardiac death (SCD) is known to be a leading cause of death in sporting participants generally,[12, 13] and specifically from results in previous AF-research.[14–16] A substantial body of research into SCD exists, particularly in understanding the underlying causes into why/how SCD occurs in sporting populations,[17] preventing SCD in athletes through screening[18–20] as well as the acute management of cardiac arrest in sports settings.[21] This focus on prevention and management is underpinned by data from studies conducted mainly in the United States and Europe.[22–24] The rates presented within those studies vary substantially owing to the type of data collection (e.g. prospective, retrospective), source of data (e.g. media, death certificates), study setting (e.g. general population, military-only) and the population included (e.g. all ages, children-only). There are no similar publications from a broad Australian sports perspective to identify the number of cases of cardiac-related deaths in sport.[25] One Australian report of data from an annual community running event identified nine cases of SCD in 25 years of the event’s history [26] and a second study reported on 14 cases of sudden death in AF using coronial data for one state (Victoria)[16]. Some of the factors that have been identified as increasing the risk of SCD add further support to the need to collect and report the number of cases of SCD for Australian sports participants specifically. As an example, research from the USA has identified higher risks of SCD in the college setting for males, African-Americans and basketball players.[27] Strong genetic links, in combination with different sports participation behaviours and health services in Australia, would suggest a different incidence and risk for such events is likely. Certainly there is a possibility for an increased risk of SCD in sport for Indigenous Australians.[15] With current support for the acute management of SCD being promoted through the recommendation and delivery of automated external defibrillators in Australia,[28, 29] establishing routine collection of SCD data would provide important information to evaluate the impact of newly introduced treatment/management initiatives on reducing fatalities from sports-related sudden cardiac arrest.

Our study reported no identifiable sport participation related injury deaths (e.g. from a hit causing damage to brain / internal organ as has been reported in other sports [1]). However, previous research has reported injuries as a cause of death specific to AF, with two in-hospital injury-related deaths between 2011–2012 due to incidents whilst playing AF identified in a larger investigation of all national sports related hospitalisations.[30] No details on the cause of these deaths was reported. Possible reasons for why these injury-related deaths were not identified in our study (despite overlap in the study time frames) include: they were in our unconfirmed/other group of causes because of the lack of detail in the descriptive narrative; they were not covered by insurance or the next of kin did not file an insurance claim; or they were participating in AF activities outside of a registered club setting that were not covered by insurance.

Injury deaths in Australian sport have also previously been investigated using data from the Victorian State Trauma Registry (VSTR) and the National Coroners’ Information System (NCIS) from 1 July 2001–30 June 2007 in the state of Victoria.[31, 32] In those studies (which partly covered overlapping time periods as our data), no injury deaths were reported for AF.[31, 32] However, injury-deaths for AF from those data sources may also be underestimated due to the way that data are collected and coded. The VSTR includes information from all patients admitted to state trauma system health services (public and private) in Victoria, and the NCIS is a national, centralised database of coronial information and includes information on all cases of unexpected death, irrespective of the cause. For hospital datasets, coded to the International Classification for Diseases, the ‘activity at time of event’ may be missing, or coded as an ‘unspecified activity’ in which AF-related cases cannot be identified.[33–35] Large numbers of ‘unidentified’[33, 34] or ‘other specified activity’[35] cases have been reported in sports injury studies more broadly and it has been estimated that up to 20% of sports injuries may not be identifiable in hospital data collection due to under-coding of activity.[33] Therefore, it appears that to obtain data relating to on-field injury deaths direct capture through dedicated sports injury surveillance is required.

Aside from the direct-injuries previously explored in AF-research,[30–32] there are also additional causes of death that may have contributed to the unconfirmed/other cases of on-field deaths. Based on leading causes of sports-related deaths in the US, it might be expected to identify cases of injury (particular to the brain or spinal cord), heat-stroke or asthma.[3] However, without more detailed data on the cause of death, it is currently not possible to ascertain what these other causes may be in specific relation to AF or to identify the management and prevention measures that might be used to counter these incidents. Certainly, there is cause to call for more coordinated efforts toward reporting this information.

### Off-field deaths

Although more than half of the cases occurred during an on-field incident, the leading single identifiable cause of death in this study was road trauma, involving motor vehicle or bicycle crashes. Other studies have also reported high proportions of road traffic deaths in athlete groups.[2, 36] This finding likely reflects broader population figures, with males aged 18–40 years having relatively large representation in both football/sport participation and road trauma cases.[37, 38] Although not related to participation in the sport directly, these road traffic deaths suggest a possible avenue for road safety messages to be communicated through sports clubs/teams to reach (a part of) their intended target group.

### Limitations

This is the first study in Australia to report insurance data claims for deaths occurring in a sporting context, with this insurance data adding important new findings to previous literature. However, there are limitations associated with the insurance data presented, which is still likely to underestimate AF-related deaths. A major reason for this underestimate is that filing an insurance claim is restricted to registered members of community clubs. Not everyone who is injured or has a cardiac event will lodge an insurance claim even when they are entitled to do so. A cause of death was unable to be ascertained from the insurance claim record in over half of the cases, severely limiting our ability to identify the most relevant measures for prevention. Understanding and determining the mechanism of death in AF relies on rich descriptive text of the incident within the database. For example, a large number of deaths were described as ‘collapsed’ with no underlying or exact cause of the collapse reported. All cases of sudden death of unknown origin are referred to a coroner for investigation and diagnosis, a process that can extend over a length period (i.e. many months). However, insurance claims must be lodged within 180 days, and at this time, coronial enquiries may not yet have been resolved. As such, when initiating a claim, many will not have a cause of death, as was the situation with around half of the cases in the current study. This presents a major limitation of using insurance data for routine surveillance of death in sport, unless processes to update the cause of death when known can be put in place.

## Conclusions

The current data and previous published literature on AF fatalities appears discrepant in the numbers and types of cases captured, and likely underestimates all causes of death, both on-field and off-field. Based on available current sources, it is recommended that when reporting sporting-related deaths in Australia that multiple sources of information be used. A common identifier (e.g. date of death combined with the sport) would be needed to identify incident cases and ensure no duplication of reporting.

Many sport-related deaths in Australia are potentially preventable but specific details on the events surrounding them are first required to better understand why they occurred. With an average of three fatalities per year reported for insurance claims from within community AF only, preventable fatalities need to be identified. The results of this study provide support for a systematic, comprehensive approach to data collection, to better understand the number and causes of death, not only in AF but in all sports.

## Abbreviations

AF: Australian Football
AFNRRP: Australian Football National Risk Protection Program
JLT: Jardine Lloyd Thompson
SCD: Sudden cardiac death
VSTR: Victorian State Trauma Registry
NCIS: National Coroners’ Information System
